# EMG-Based Characterization of Walking Asymmetry in Children with Mild Hemiplegic Cerebral Palsy

**DOI:** 10.3390/bios9030082

**Published:** 2019-06-27

**Authors:** Francesco Di Nardo, Annachiara Strazza, Alessandro Mengarelli, Stefano Cardarelli, Andrea Tigrini, Federica Verdini, Alberto Nascimbeni, Valentina Agostini, Marco Knaflitz, Sandro Fioretti

**Affiliations:** 1 Department of Information Engineering, Università Politecnica delle Marche, Via Brecce Bianche, 60131 Ancona, Italy; 2 Rehabilitation Unit, Ospedale S. Croce, A.S.L. TO5, P.zza A. Ferdinando 3, Moncalieri (TO), 10024 Torino, Italy; 3 Department of Electronics and Telecommunications, Politecnico di Torino, Corso Duca degli Abruzzi 24, 10129 Torino, Italy

**Keywords:** surface electromyography, cerebral palsy, hemiplegia, children locomotion, motor disorders, gait

## Abstract

Hemiplegia is a neurological disorder that is often detected in children with cerebral palsy. Although many studies have investigated muscular activity in hemiplegic legs, few EMG-based findings focused on unaffected limb. This study aimed to quantify the asymmetric behavior of lower-limb-muscle recruitment during walking in mild-hemiplegic children from surface-EMG and foot-floor contact features. sEMG signals from tibialis anterior (TA) and gastrocnemius lateralis and foot-floor contact data during walking were analyzed in 16 hemiplegic children classified as W1 according to Winter’ scale, and in 100 control children. Statistical gait analysis, a methodology achieving a statistical characterization of gait by averaging surface-EMG-based features, was performed. Results, achieved in hundreds of strides for each child, indicated that in the hemiplegic side with respect to the non-hemiplegic side, W1 children showed a statistically significant: decreased number of strides with normal foot-floor contact; decreased stance-phase length and initial-contact sub-phase; curtailed, less frequent TA activity in terminal swing and a lack of TA activity at heel-strike. The acknowledged impairment of anti-phase eccentric control of dorsiflexors was confirmed in the hemiplegic side, but not in the contralateral side. However, a modified foot-floor contact pattern is evinced also in the contralateral side, probably to make up for balance requirements.

## 1. Introduction

Hemiplegia is a neurological disorder that is frequently detected in children with cerebral palsy. It may provoke altered selective motor control, weakness, and spasticity [1]. While one side is affected by the disorder, the contralateral side appears to maintain complete functionality. The consequent asymmetry has been proven to affect many daily-life tasks, such as walking [1]. Therefore, the possibility of characterizing hemiplegic-child walking by features that are able to quantify asymmetries in lower-limb recruitment should be considered in order to describe control strategies and support clinicians and physical therapists in planning treatment approaches. From this point of view, a reliable classification of hemiplegia is fundamental [2]. An acknowledged classification was proposed by Winters et al. [3] for analyzing the most frequent walking patterns in hemiplegic children and young adults. Four different classes were distinguished, based on the progressive distal-proximal involvement of the paretic leg. Winters’ type I was characterized by hypo-activation of ankle dorsiflexors in the hemiplegic side, which elicited drop foot during the swing phase. A more severe condition was detected in Winters’ type II: persistence of equinism all through gait cycle, associated with a possible knee hyperextension in stance. Winters’ type III also presented a reduced knee flexion in swing. In addition to the previous conditions, Winters’ type IV showed a reduced motion of the hip [3].

Winters’ type I and II have been the most recurrent hemiplegic forms observed in cerebral palsy [4] and therefore have been frequently investigated [4,5,6]. In particular, a recent study [5] attempted to quantify the myoelectric activity of ankle muscles in a population of Winters’ type I and type II children during walking. Analysis of electromyographic signal has indeed, been frequently used to complement information coming from classic gait analysis [7,8,9,10]. The abovementioned study [5] focused on the hemiplegic side, revealing a large variability in muscle activation patterns. Significant differences with respect to control children were also detected [5]: A curtailed activity of tibialis anterior (TA) during terminal swing and a lack of activity at loading response in the hemiplegic side (in type I and II) and a hyper-activation of gastrocnemius around initial contact (in type II only). Modified myoelectric patterns in the hemiplegic leg would be expected to determine compensatory alterations in the recruitment of contralateral leg muscles. Nevertheless, few EMG-based findings have been reported on the unaffected limb during walking task [9,11,12,13].

The goal of the current analysis was the quantification of asymmetric behavior of ankle-muscle recruitment during walking in type I hemiplegic children according to Winters’ classification. Ankle muscles were selected because the definition of Winters’ type I hemiplegia focused on these muscles [3]. Asymmetry was quantified not only in terms of classical features extracted from surface electromyography (sEMG), such as onset/offset instants of activation, but also in terms of more recent parameters, such as the activation modality (which defines the number of times a single muscle activates during a single gait cycle) and the occurrence frequency (defined as the frequency each muscle activation occurs with). Analysis of foot-floor contact sequence and gait-phase duration was used to support sEMG data. To our knowledge, this is the first study that has tried to assess gait asymmetry in hemiplegic children in terms of activation modality and occurrence frequency of muscular recruitment. Moreover, the present study is the first to quantify EMG-based gait asymmetry separately for each foot-floor contact class. A large number of gait cycles were included in the analysis (around two hundred and fifty cycles for each child) in order to handle the expected variability of hemiplegic walking.

## 2. Materials and Methods

### 2.1. Subjects

Gait data from hemiplegic and control children were taken from retrospective studies performed at Laboratory of Gait Analysis, Ospedale Santa Croce, Moncalieri (TO), Italy [5,14]. The Laboratory database was searched for children aged 5 t o13 years with Winters’ group I hemiplegia. Children who underwent previous lower limb orthopedic surgery or botulinum toxin injections in the six months preceding the gait examination were excluded from the study. Two raters independently examined the video recordings and kinematic data, and selected a total of sixteen hemiplegic children (Winters’ group I (W1), age range: 5–13 years; 10 males/6 females; 11 right/5 left hemiplegia; age: mean 8.9, SD 2.8 years; height: mean 132, SD 16 cm; mass: mean 29.3, SD 9.9 kg [5]). One hundred able-bodied children (age range: 6–11 years; 51 males/49 females; age: mean 9.0, SD 1.4 years; height: mean 133, SD 9 cm; mass: mean 30.6, SD 6.7 kg [14]) were included as controls. Data were analyzed comparing three different groups.

HS-group (Hemiplegic-Side group, *n* = 16). This group was created to characterize the behavior of the hemiplegic leg of hemiplegic children. This group included all of the 16 hemiplegic children recruited for this study. In this group, basographic and myoelectric data from the hemiplegic side of each child were analyzed.

CS-group (Contralateral-Side group, *n* = 16). This group was created to characterize the behavior of the contralateral (non-hemiplegic) leg of hemiplegic children. This group included the same children as the HS-group. However, in this group basographic and myoelectric data from the contralateral (non-hemiplegic) side of each child were analyzed.

CON-group (Control-children group, *n* = 100). This group included 100 able-bodied children, who were considered as control children. In this group, basographic and myoelectric data from both sides of the control children were analyzed.

The present research was undertaken following the ethical principles of Helsinki Declaration and was approved by local ethical committee.

### 2.2. Signal Acquisition

Surface EMG signals were recorded (sampling rate: 2 kHz; resolution: 12 bit) by the multichannel recording system Step32 (Medical Technology, Turin, Italy). To this aim, single differential sEMG probes (Ag-disks; diameter 4 mm; inter-electrode distance 12 mm; gain 1000; high-pass filter 10 Hz, 2 poles) were positioned on TA and GL bilaterally following Winter’s guidelines [15]. Three foot-switches (10 × 10 mm, thickness 0.5 mm, activation force 3 N) were applied under the heel and on the first and the fifth metatarsal heads of each foot for measuring foot-floor contacts. Then, children were requested to walk barefoot back and forth over a 10-m straight walkway at their self-selected speed and cadence for approximately 2 min and 30 s. Crosstalk was checked for by visual inspection. Crosstalk was suspected when two muscles in the same limb section showed simultaneous activity with similar amplitude modulation. In this case, double differential probes were used to further improve spatial selectivity. The double differential signal was compared with the single differential signal. If the amplitude of the double differential signal was significantly lower, crosstalk was confirmed and the signal was discarded. Double differential probes were three-bar probes (bar diameter: 1 mm, bar length: 10 mm, interelectrode distance: 10 mm) with gain and filtering properties equal to those of the single differential probes.

### 2.3. Signal Processing

sEMG signals were band-pass filtered (20–450 Hz). Myoelectric activation intervals were identified by means of a double-threshold statistical detector [16]. This methodology [16] consists of selecting a first threshold ζ and watching *m* successive samples: if at least *r_0_* (second threshold) out of successive *m* samples are above ζ, the presence of the signal is acknowledged. Values of the three parameters ζ, *r_0_*, and *m* are selected to jointly minimize the false-alarm probability value and maximize the detection probability for each specific signal-to-noise ratio. The setting of ζ is based on the estimation of background noise level, as a necessary input parameter. Furthermore, the double-threshold detector requires that the signal-to-noise ratio is estimated in order to fine tune *r_0_*. Background noise level and signal-to-noise ratio, which are necessary in order to run the double-threshold algorithm, were assessed for every signal by the Step32 system, using the statistical approach proposed by Agostini and Knaflitz [17]. Finally, *m* = 30 ms is considered a suitable value for evaluating muscle activation in gait analysis [16]. Further details of the algorithm description can be found in a study by Bonato et al. [16]. A 4-level signal was extracted from the three binary foot-switch signals [18], corresponding to the following gait phases: Heel strike phase (H): Only the foot-switch under the heel is closed; Flat foot contact phase (F): The foot-switch under the heel is closed and at least one of the foot-switches under the forefoot is also closed; Push off phase (P): The foot-switch under the heel is open and at least one of the foot-switches under the forefoot is closed; and Swing phase (S): All foot-switches are open. Then, foot-switch signals were processed to identify each gait cycle (GC) [18]. The sequences of gait phases observed in the foot-floor contact analysis were indicated as follows: HFPS was the normal sequence of gait phases characterized by the following foot-floor contact sequence: Heel contact, flat-foot contact, push-off, swing); PFPS was characterized by a forefoot initial-contact (P1), then the heel also touched the floor (F), and P and S phases followed; PS started with forefoot contact (P), followed immediately by swing phase (S), With the heel never touching the ground. Further details on signal acquisition and processing can be found in a study by Agostini et al. [14].

### 2.4. Statistical Gait Analysis

In this study, human walking was characterized by means of average sEMG features and spatial-temporal parameters extracted from hundreds of consecutive strides for each child. A recent approach was adopted to this aim [14,19], based on the cycle-dependency of muscular activation during walking task. sEMG parameters were averaged over those cycles including the same number of activations (i.e., over each single activation modality). The activation modality defines the number of times a muscle activates during a single gait cycle: the *n*-activation modality consists of *n* active intervals for the considered muscle during a single gait cycle. To provide average intervals of muscle activation for each modality, onset/offset instants were computed in every gait cycle [16]. Muscle activations were then gathered according to the number of detected intervals (i.e., relative to their activation modality). Eventually, onset/offset instants were averaged over the entire population for each activation modality and normalized with respect to gait-cycle duration. The very large number of strides considered in the analysis allowed for the assessment of a new parameter, known as occurrence frequency [20,21]. The occurrence frequency of a specific activation modality is quantified by the number (in percentage) of strides where the muscle is recruited with this specific activation modality, with respect to the number of total strides, as reported in the following formula:

$$Occurrence\;Frequency\;\left(n\right)=\frac{Number\;of\;gait\;cycles\;with\;n\;activation\;intervals}{Total\;number\;of\;gait\;cycles}\times 100$$

Statistical gait analysis was performed by means of the statistical toolbox embedded in the Step32 system for gait analysis.

### 2.5. Statistics

Data were reported as mean ± standard error (SE). The Shapiro–Wilk test was used to evaluate the normality of each distribution. ANOVA and Kruskal–Wallis tests were used to compare normally and non-normally distributed samples, respectively. Statistical significance was set at 5%.

## 3. Results

A total of 4182 strides in the hemiplegic children and more than 30,000 strides in control children were analyzed, involving only HFPS, PFPS, and PS sequences of foot-floor contacts.

### 3.1. Foot-Floor Contact

Three main foot-floor contact sequences were detected: HFPS, PFPS, and PS. Their mean percentages were quantified and compared in all three of the groups. For the HFPS sequence, the percentage was significantly (*p* = 3.10 × 10^−7^) lower in the HS-group (35 ± 9%) with respect to the CS-group (79 ± 3%) and the CON-group (88 ± 1%). For the PFPS sequence, the percentage was significantly (*p* = 2.23 × 10^−4^) higher in the HS-group (44 ± 8%) with respect to the CS-group (8 ± 1%) and the CON-group (5 ± 0.5%). No significant differences (*p* > 0.05) were detected among groups for the PS sequence: HS-group = 3.0 ± 2.0%, CS-group = 1.0 ± 0.3%, and CON-group = 1.0 ± 0.1%. The CS-group also presented 11 ± 6% of strides with a sequence starting with the P-phase, but different from PFPS and PS. These sequences were negligible in the other groups. Due to the low percentage of their occurrence, data from the PS sequence were not analyzed.

**Figure 1:**
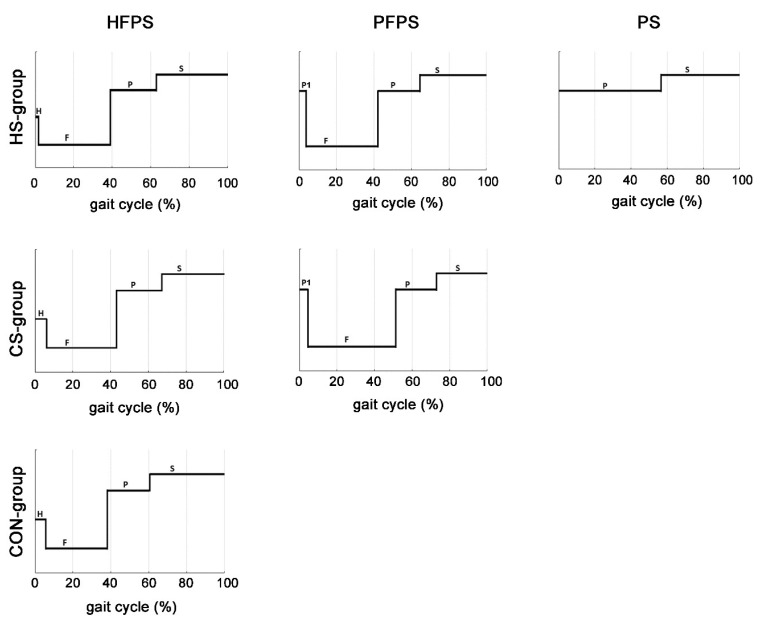
Foot-floor contact sequences and gait-phase timing.

Average gait-phase timing associated with the main foot-floor contact sequences is presented in Figure 1 for the HS-group (HFPS, PFPS, and PS foot-floor contact sequences, upper panel), the CS-group (HFPS and PFPS sequences, middle panel), and the CON-group (HFPS sequences, lower panel), respectively. Some significant differences in timing were found among the three groups. For the HFPS sequence, there was a significant reduction (*p* = 1.19 × 10^−7^) of the H-phase in the HS-group (1.8 ± 0.2% of gait cycle) compared to the CS-group (5.9 ± 0.6%) and the CON-group (5.8 ± 0.1%); a significant reduction (*p* = 3.17 × 10^−7^) of the S-phase in the CS-group (32.9 ± 1.1%) compared to the HS-group (37.1 ± 2.5%) and the CON-group (39.6 ± 0.3%); and a significant increase (*p* = 3.36 × 10^−7^) of the whole stance phase in the CS-group (67.1 ± 1.1%) compared to the HS-group (62.9 ± 2.5%) and the CON-group (60.4 ± 0.3%). For the PFPS sequence, there was a significantly shortened initial toe-contact (P1-phase) and F-phase in the HS-group (3.8 ± 0.6% and 38.1 ± 2.6%, respectively) compared to the CS-group (4.7 ± 0.5%, *p* = 1.00 × 10^−5^ and 46.4 ± 2.4%, *p* = 2.05 × 10^−2^, respectively); a significantly shortened S-phase in the CS-group (27.3 ± 1.4%) compared to the HS-group (35.8 ± 1.9%, *p* = 2.38 × 10^−4^); and a significant increase (*p* = 2.41 × 10^−4^) of the whole stance phase in the CS-group (72.7 ± 1.3%) compared to the HS-group (64.2 ± 1.9%). The CON-group was not involved in this comparison because the number of PFPS sequences detected was negligible.

### 3.2. Muscle Activation Patterns

**Figure 2:**
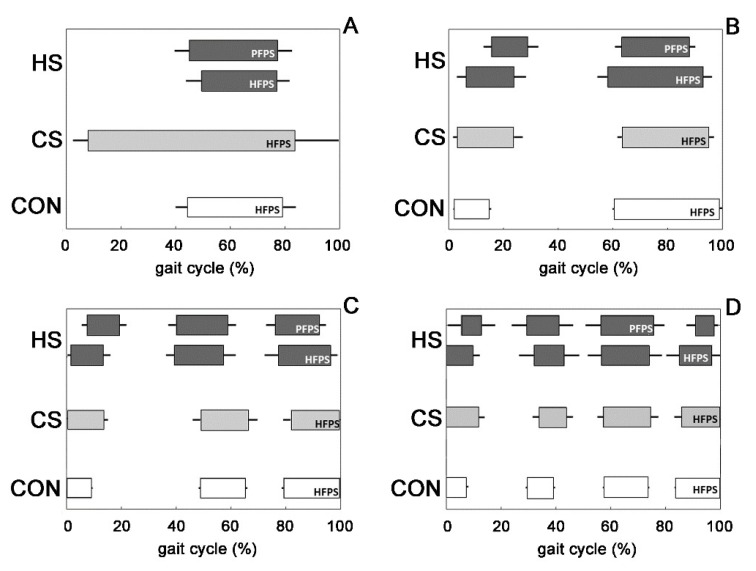
Tibialis anterior (TA) activation intervals in the main four modalities of activation in the HS-group (**dark gray**), the CS-group (**light gray**), and the CON-group (**white**).

The average muscular activation intervals related to the main four activation modalities for TA are shown in Figure 2 for the HS-group (dark grey bars), the CS-group (light grey bars), and the CON-group (white bars), respectively. For the HS-group, activation intervals were reported separately for HFPS and PFPS foot-floor contact sequences. The activation intervals were expressed in function of percentage gait cycle. TAi (i = 1–5) are the modality of TA activation with one activation (TA1, Figure 2A), two activations (TA2, Figure 2B), three activations (TA3, Figure 2C), four activations (TA4, Figure 2D), and five activations (not shown because not statistically relevant) during a single gait cycle. In this section and in the following section, data from PFPS sequences are not shown for the CS-group and the CON-group due to their infrequent occurrence. Some main differences in TA muscular recruitment were detected among the three groups: a significant delay of TA onset in the PFPS sequence of the HS-group for the modalities TA1 (*p* = 1.39 × 10^−14^, Figure 2A) compared to the CS-group, and TA2 (*p* = 2.80 × 10^−9^, Figure 2B), and TA3 (*p* = 3.72 × 10^−12^, Figure 2C), compared to both the CS-group and the CON-group; a significantly earlier TA offset in the PFPS sequence of the HS-group for the modalities TA2 (*p* = 5.12 × 10^−8^, Figure 2B) and TA3 (*p* = 2.56 × 10^−13^, Figure 2C), compared to both the CON-group and the CS-group.

**Figure 3:**
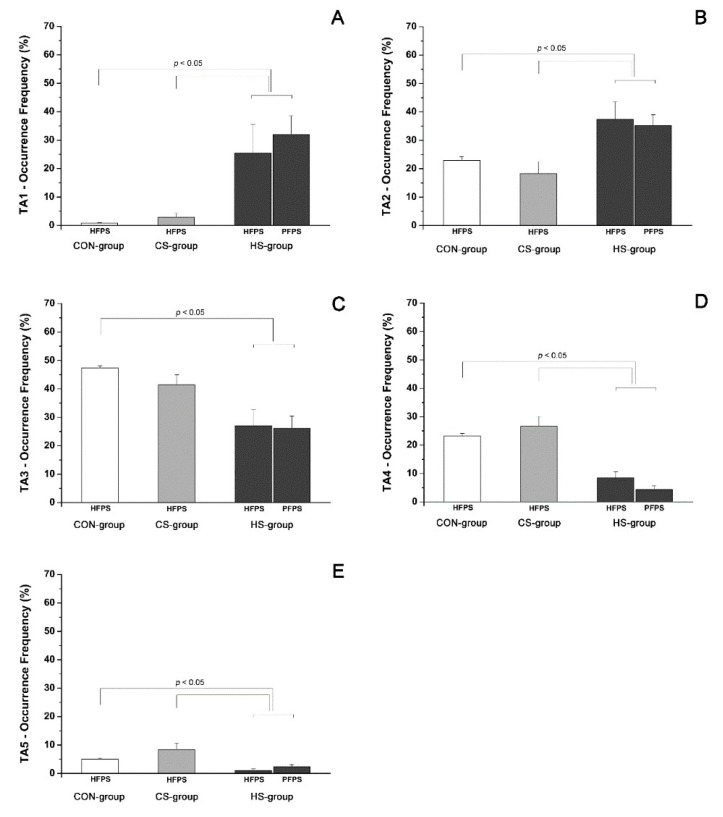
Occurrence frequency of TA recruitment in the HS-group (**dark gray**), the CS-group (**light gray**), and the CON-group (**white**) for every activation modality.

The average occurrence frequencies of muscular recruitment related to all of the detected activation modalities for TA are shown in Figure 3 for the CON-group (white bars), the CS-group (light grey bars), and the HS-group (dark grey bars), respectively. For the HS-group, occurrence frequencies are reported separately for the HFPS and PFPS foot-floor contact sequences. Some significant differences (*p* < 0.05) in occurrence frequency of TA muscular recruitment were detected among the three groups and are highlighted in Figure 3 by horizontal square brackets. The most frequent modality of activation for TA changed among groups: it was a 2-activation modality both for HFPS (TA2, 37.4 ± 6.1% of total strides) and PFPS (TA2, 35.3 ± 3.8%) sequences of the HS-group and a 3-activation modality for both the CON-group (TA3, 47.3.4 ± 0.8%) and the CS-group (TA3, 41.4 ± 3.5%). A significant increase of the occurrence frequency (for both the HFPS and PFPS sequences) was observed in the HS-group for the modalities TA1 (Figure 3A, *p* = 2.13 × 10^−14^) and TA2 (Figure 3B, *p* = 2.01 × 10^−3^), compared to both the CON-group and the CS-group. A concomitant significant reduction of the occurrence frequency (for both the HFPS and PFPS sequences) was observed in the HS-group compared to (1) the CON-group for the modalities TA3 (Figure 3C, *p* = 1.23 × 10^−7^), TA4 (Figure 3D, *p* = 1.64 × 10^−10^), and TA5 (Figure 3E, *p* = 5.10 × 10^−5^); and (2) the CS-group for the modalities TA4 (Figure 3D, *p* = 1.64 × 10^−10^) and TA5 (Figure 3E, *p* = 5.10 × 10^−5^). On the contrary, no significant differences (*p* > 0.05) were detected between the CS-group and the CON-group in the evaluation of the occurrence frequency for all the modalities of TA activation.

**Figure 4:**
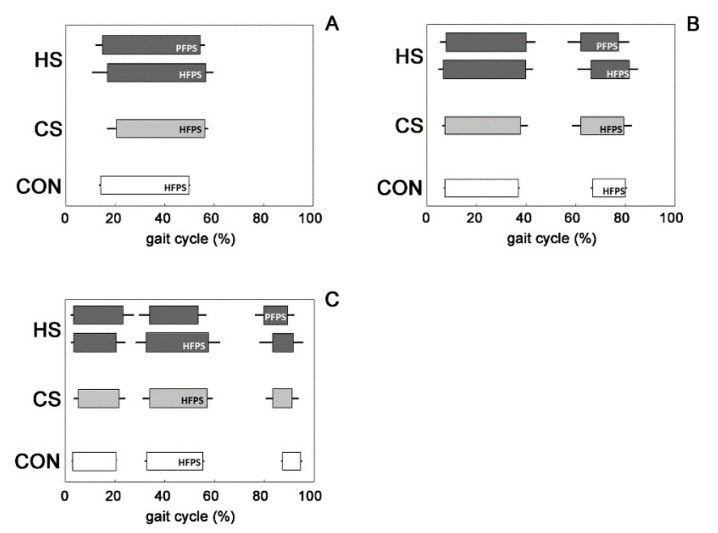
GL activation intervals for the main three modalities of activation in the HS-group (**dark gray**), the CS-group (**light gray**), and the CON-group (**white**).

**Figure 5:**
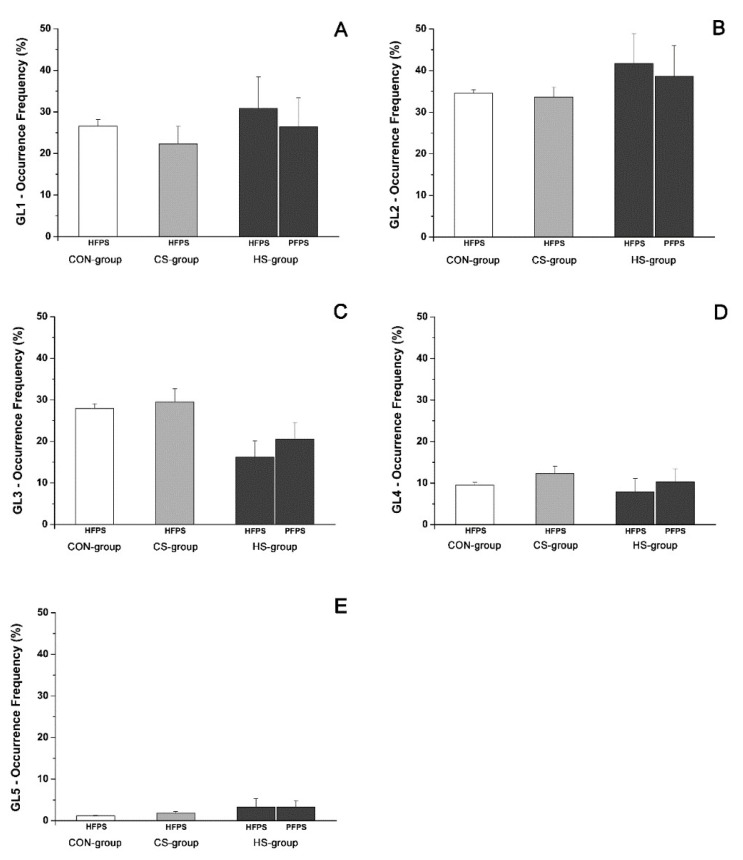
Occurrence frequency of GL recruitment in the HS-group (**dark gray**), the CS-group (**light gray**), and the CON-group (**white**) for every activation modality.

The average muscular activation intervals related to the main three activation modalities for GL are shown in Figure 4 for the HS-group (dark grey bars), the CS-group (light grey bars), and the CON-group (white bars), respectively. For the HS-group, activation intervals are reported separately for the HFPS and PFPS foot-floor contact sequences. The activation intervals are expressed in function of percentage gait cycle. Average occurrence frequencies of muscular recruitment related to all of the detected activation modalities for GL are shown in Figure 5 for the CON-group (white bars), the CS-group (light grey bars), and the HS-group (dark grey bars), respectively. For the HS-group, occurrence frequencies are reported separately for the HFPS and PFPS foot-floor contact sequences. GLi (i = 1–5) are the modality of GL activation with one activation (GL1, Figure 4A), two activations (GL2, Figure 4B), three activations (GL3, Figure 4C), four activations, and five activations (not shown because not statistically relevant) during a single gait cycle. The three groups did not show significant alterations (*p* > 0.05) in mean activation intervals (Figure 4) among groups and mean occurrence-frequency values (Figure 5) for all of the activation modalities; moreover, the most frequent modality of activation remained the same among groups (2-activation modality, Figure 5B).

## 4. Discussion

This work was designed to study asymmetries in hemiplegic-children walking, by means of an automatic analysis of gait data acquired during around two hundred and fifty cycles for each child. Only hemiplegic children, acknowledged as group I (W1) in the classification of spastic hemiplegia introduced by Winters et al. [3], were considered.

### 4.1. Foot-Floor Contact

The analysis of foot-floor contact sequences showed that the first contact of the hemiplegic foot with the floor was with the heel in 35% of the strides and with the forefoot in 58% of the strides. The remaining 7% of the strides were characterized by different contacts (F), which were not statistically significant. Even though the forefoot contacts seem to outnumber the rest, a single standard behavior cannot be identified, and the two main patterns (HFPS and PFPS) alternated. Based on the analysis of a few gait cycles, gait analysis is used to characterize hemiplegic walking with only forefoot initial-contact cycles [22]. The present study, performed with hundreds of strides for each child, indicated that a reliable approach should also consider strides with heel initial-contact and not only strides with and forefoot initial-contact. Differently from the hemiplegic side, the contralateral foot landed with the heel in 79% of the strides, indicating that HFPS was the most prevalent foot-floor contact scheme, as in the control children (88%). This showed that in the contralateral leg the heel-rocker is generally preserved, as also the case in the strides where hemiplegic foot adopted a forefoot strike. It is interesting to note that the F-phase was preserved (PS sequence <3%) in both the hemiplegic and contralateral sides.

The analysis of temporal gait parameters is commonly limited to the instants of initial contact and toe-off [23,24] when assessing the two main gait phases: Stance and swing [9]. This is even truer in hemiplegic walking, where the detection of the foot contact event is particularly challenging due to forefoot initial contact [23]. Nevertheless, this information could not be sufficient for discriminating different pathological walking or different stages in the evolution of a specific pathology, such as hemiplegia [5]. Thus, in the present study, the detection of the foot-floor contact event was extended to three sub-phases of the stance: heel strike, flat-foot contact, and push-off for HFPS sequence and initial toe-contact, flat-foot contact, and push-off for PFPS sequence. A shorter stance phase was detected in the hemiplegic side vs. the contralateral side for both the HFPS and PFPS sequences (Figure 1). This is mainly due to the reduction of the initial-contact sub-phase (H in HFPS and P1 in PFPS) in the hemiplegic side with respect to the contralateral side. A consequent increment of swing phase was noticed. Moreover, stance phase was longer in the contralateral side also with respect to control subject, matching with the results of Agostini et al. [5]. This confirms that in the hemiplegic children the balance maintenance during walking is accomplished mainly by the contralateral “healthy” foot.

### 4.2. Muscle Activation Patterns

The recruitment of ankle muscles in hemiplegic children presented a large variability in terms of the number and timing of activation intervals in both lower limbs. Similar results were reported for able-bodied children [14]. The typical activation of tibialis anterior started just before toe-off and full swing activity, continuing up to initial stance in both adults and children [14,25]. The present results on able-bodied children matched with this description. Otherwise in hemiplegic children, a significant (*p* < 0.05) lack of TA activity was detected in the affected side around initial contact only for the strides adopting PFPS foot-floor contact sequence, in line with the definition of group I introduced by Winters [3]. Absence of TA activity, highlighted in Figure 2 by the delay of the initial activity and a concomitant curtailed activity during terminal swing, was statistically significant (*p* < 0.05) and visually more evident in the modalities characterized by a lower number of activations (Figure 2A,B). These modalities were the most recurrent ones (*p* < 0.05) within the HS-group (Figure 3) and more recurrent than the corresponding modalities of the CS-group and the CON-group (Figure 3A,B), which seemed to prefer adopting modalities with a higher number of activations. This led to identifying that the lack of TA activity occurred significantly (*p* < 0.05) in 93.3 ± 4.6% of PFPS strides (i.e., around 50% of total hemiplegic strides (PFPS + HFPS)). The phenomenon was not observed in the contralateral side (Figure 2). This means that the impairment of anti-phase eccentric control of the dorsiflexors reported previously for hemiplegic children [3] was confirmed here only in PFPS strides of hemiplegic side, but not in HFPS strides. The contralateral leg seems not to be (or only slightly) involved in this impairment process. The modification of activations patterns and the consequent asymmetry between legs involved only TA. No significant differences were detected in GL activation intervals (Figure 4) and occurrence frequencies (Figure 5) among the groups, indicating the absence of relevant variation of muscular recruitment between the hemiplegic and non-hemiplegic side for plantar flexors, represented by GL. Results on GL were preliminary reported in Di Nardo et al. [26].

One of the novelties of the present study was the assessment of the activation modalities and occurrence frequency to quantify asymmetric behavior in hemiplegic children. The analysis of these parameters could be useful for quantifying how often a child exploits a specific gait pattern, how much the pattern is different between legs, and, consequentially, to what extent this pattern is representative of child gait. This also supplies information on patient intra-subject variability, an important aspect to be considered in longitudinal follow-up. Moreover, the presence of several distinct patterns of foot-floor contact and EMG activation in the same Winters’ type may be used as a new or at least a supplementary classification for distinguishing hemiplegic cerebral palsy CP (cerebral palsy) children, possibly helping in the personalization of therapeutic care or in intervention decision-making.

This study exercised special care in recruiting subjects belonging to a specific hemiplegic class (W1) and to a specific age range (school-age children). Our restrictive selection reduced the number of patients eligible for the study but strengthened the reliability of results.

## 5. Conclusions

The present study was able to quantify the asymmetric behavior of mild hemiplegic children during self-selected walking, by assessing foot-floor contact sequence/duration and features characterizing sEMG signal. To our knowledge, this was the first attempt to quantify this asymmetry in terms of activation modality and the occurrence frequency of the muscular recruitment separately for each foot-floor contact class. This approach highlighted different aspects of hemiplegic walking, allowing us to (1) quantify the asymmetry in dorsiflexor recruitment due to the reduced TA activity around initial contact in the hemiplegic side; (2) assess that the above-mentioned asymmetry did not occur in all of the walked strides, but only in 50% (i.e., the strides characterized by a PFPS foot-floor contact sequence); and (3) identify an asymmetry in spatial/temporal parameters due to stance phase prolongation in the contralateral (non-hemiplegic) side in the HFPS sequence, also with respect to control subjects, suggesting the need for modified foot-floor contact patterns also in the contralateral side, probably to make up for balance requirements.
